# Alternative negative weight for simpler hardware implementation of synapse device based neuromorphic system

**DOI:** 10.1038/s41598-021-02176-4

**Published:** 2021-12-01

**Authors:** Geonhui Han, Chuljun Lee, Jae-Eun Lee, Jongseon Seo, Myungjun Kim, Yubin Song, Young-Ho Seo, Daeseok Lee

**Affiliations:** Department of Electronic Materials Engineering, Kwangwoon University, Seoul, 01897 Republic of Korea

**Keywords:** Engineering, Nanoscience and technology

## Abstract

Lately, there has been a rapid increase in the use of software-based deep learning neural networks (S-DNN) for the analysis of unstructured data consumption. For implementation of the S-DNN, synapse-device-based hardware DNN (H-DNN) has been proposed as an alternative to typical Von-Neumann structural computing systems. In the H-DNN, various numerical values such as the synaptic weight, activation function, and etc., have to be realized through electrical device or circuit. Among them, the synaptic weight that should have both positive and negative numerical values needs to be implemented in a simpler way. Because the synaptic weight has been expressed by conductance value of the synapse device, it always has a positive value. Therefore, typically, a pair of synapse devices is required to realize the negative weight values, which leads to additional hardware resources such as more devices, higher power consumption, larger area, and increased circuit complexity. Herein, we propose an alternative simpler method to realize the negative weight (named weight shifter) and its hardware implementation. To demonstrate the weight shifter, we investigated its theoretical, numerical, and circuit-related aspects, following which the H-DNN circuit was successfully implemented on a printed circuit board.

## Introduction

Recently, there has been a substantial increase in the consumption of unstructured data, such as images, movies, songs, sensory signals, and others^1,2^. To effectively analyze this data, software-based deep learning neural networks (S-DNN) are widely utilized^3^. However, in hardware respect, the conventional Von-Neumann structural computing system has insufficient analog-support structure for the S-DNN, due to several inherent limitations such as high energy consumption, low data processing speed, and etc^4–6^. Thus, there is an urgent need for human–brain-inspired, potent, and efficient computing systems^7^. As one of the promising approaches, synapse-device-based hardware DNN (H-DNN) has been proposed to overcome the limitations^8,9^.

In contrast to the S-DNN, negative weight which is one of core values in the deep learning neural network (DNN) cannot be directly implemented in the H-DNN. Considering that weight is normally represented as conductance of the synapse device which always has a positive value, a pair of synapse device is employed to express the negative weight: named as pair-synapse method^10,11^. Conductance values of the two synapse devices are subtracted from each other through additional circuitry to indirectly express the negative weight, which requires additional devices, larger area, higher power consumption, and increased circuit complexity^12–14^. Hence, in this research, we propose a simpler way to implement the negative weight.

During a vector matrix multiplication (VMM) of the DNN, each weight (conductance) is multiplied to input bias, then all output currents are summed through column line (bit line)^15–20^. Because the weight can have positive or negative values in the S-DNN, the summed output currents can increase or decrease. To hardware implement the VMM, in a typical way, the negative weight value is expressed by the pair-synapse method^10,11^. In other words, conductance of one synapse device plays a role of reference conductance while conductance of the other synapse device is subtracted from the reference conductance. In the pair-synapse method, each weight needs to be expressed as two synapse devices with additional circuits; the negative weight is implemented at device level.

However, we can simply implement the negative weight by utilizing the summed output current. Based on the fact that all weights are multiplied to input bias and summed through column lines, we can make references for each output current of column line (named as weight shifter). Thus, for each column, the reference output current can be subtracted from the summed output current. In the array aspect, the negative weight can be implemented as the output current of bit line through the weight shifter. We can implement the H-DNN with less synapse devices, simple circuits, and low power consumption.

## Results and discussion

**Figure 1:**
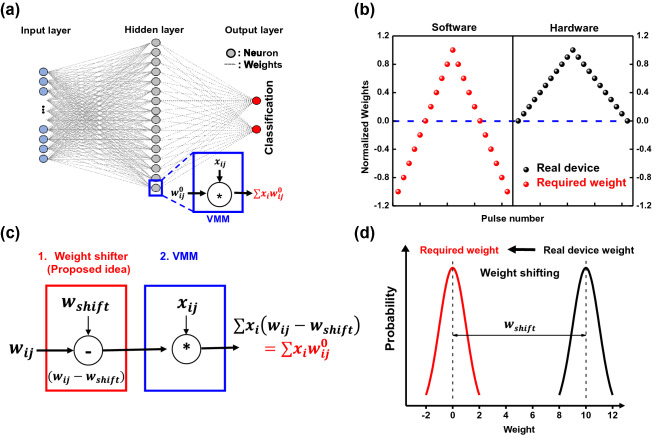
(**a**) Simple illustration of employed deep learning neural network (DNN) with the vector matrix multiplication (VMM). (**b**) Normalized required weight values in software based DNN (S-DNN), and weight values of real synapse device. Contrast to the S-DNN, the synapse device has only positive conductance values which is expressed as weight values. (**c**) Concept of proposed weight shifter to realize negative weight in the hardware based DNN (H-DNN). The positive conductance values of synapse device can be considered as positively shifted weight values. The difference between required weight values of the S-DNN and positively shifted weight values of the H-DNN (w$_{shift}$) can be subtracted during the VMM. (**d**) Distributions of weight values in the S-DNN and H-DNN. The w$_{shift}$ can be compensated by the weight shifter during VMM.

To realize the DNN, the VMM is necessary, as shown in Fig. 1a. Therefore, in the H-DNN, arrays of synapse device are utilized as the VMM. However, the negative weight can not be implemented efficiently because the conductance of synapse device is always a positive value (Fig. 1b). In other words, the S-DNN has both positive and negative weight values (±w$_{ij}^0$), and they are multiplied to input data (x$_{ij}$). Then, the multiplied results (±w$_{ij^0}$x$_{ij}$) are summed in parallel through the VMM^21–23^. Based on the ±w$_{ij}^0$ of S-DNN, final values could be increased or decreased. In contrast to the S-DNN, the synapse device can express only positive weight values. Thus, in typical way, conductance values of two synapse devices are subtracted for expression of one negative weight^12,24^. This method consumes more power and requires various additional resources, such as additional synapse devices and subtraction circuits^10^.

**Figure 2:**
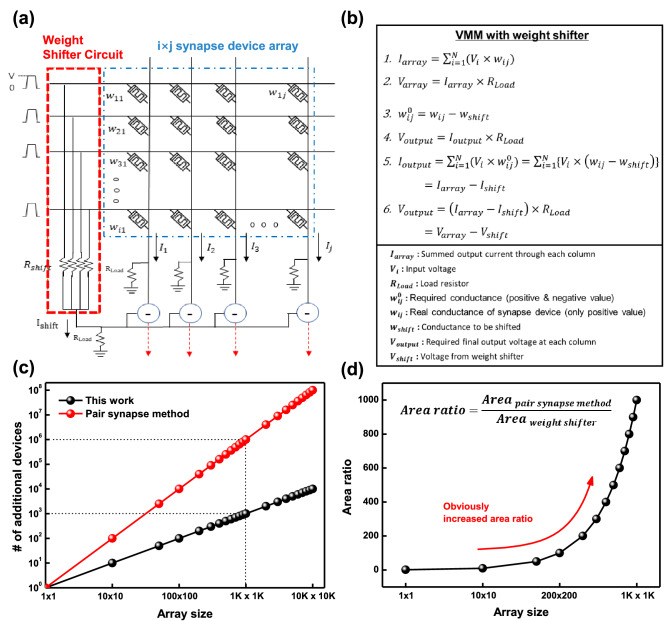
(**a**) Schematic of the weight shifter with i$\,\times \,$j synapse-device array. (**b**) Theoretical equation of the weight shifter. The conductance value of synapse device (w$_{ij}$) can be considered as positively shifted weight value ($=$w$_{ij}^0 +$w$_{shift}$). When we consider that output results of the VMM will be total columnar current (I$_{array}$), it is derived from multiplication between input data (V$_{i}$) and conductance of synapse device (w$_{ij}$). Then, output voltage of one column (V$_{array}$) can result from multiplying the I$_{array}$ by load resistor (R$_{Load}$). In the same manner, output voltage of the weight shifter can be derived by multiplying output current of the weight shifter (I$_{shift}$) by R$_{Load}$. As a result, the final output voltage of the VMM (V$_{output}$) can be obtained by subtracting the V$_{shift}$ from V$_{array}$. (**c**) Comparison of additionally required devices between conventional pair synapse method and proposed weight shifter for realization of the negative weight. (**d**) Area ratio (= area of conventional method/area of weight shifter) depending on array size.

Therefore, as shown in Fig. 1c, d, we proposed the weight shifter; the median of weight values in the S-DNN is moved from zero to positive region to make all weight values become positive. Based on the positive weight values of the S-DNN, it can be directly expressed by the conductance of synapse devices (w$_{ij}$). In this case, because all weight values are positive, the output current will be increased. Therefore, the changed value of median should be considered; we returned the median back to its original value in output current respect.

As an example, in Fig.1d, when the weight values of the S-DNN are in the range from − 2 to 2, we can shift the median of the weight values from 0 to 10. Then the weight values are in the range from 8 to 12; the positive weight values (8–12) of the S-DNN can be directly expressed by the conductance values of synapse devices. Based on these values, the VMM can be conducted through the synapse-device array. After the VMM, the shifted median of weight value needs to be returned back to 0. Thus, the difference between the original and shifted median of weight values (w$_{shift}=10$) needs to be subtracted from the VMM results (output current at each column).

The simplified H-DNN circuit with the proposed weight shifter and theoretical equation of the weight shifter are presented with details in Fig. 2a, b, respectively. Based on the input pulses (indicating input data: v$_{i}$) and synapse devices having various positive conductance values (w$_{ij}$), we can obtain results of the VMM as output current (I$_{array}$). And it leads to the output voltage (V$_{array}$) across the load resistor (R$_{Load}$); note that the w$_{ij}$ implies conductance of the synapse device, which has only positive values.

During this process, the w$_{ij}$ can be considered as already positively shifted weight because the employed conductance values are all positive. Thus, we can drive the required conductance value (w$_{ij}^0$) which is representing the weight value of S-DNN as below.

$$\begin{aligned} w_{ij}^0 = w_{ij} - w_{shift} \end{aligned}$$

Then, required output voltage of each column (V$_{output}$) can be derived from output current (I$_{output}$) and R$_{Load}$. Because the w$_{ij}^0$ is defined by w$_{ij}$ and w$_{shift}$, the I$_{output}$ is derived as I$_{array}$ − I$_{shift}$. Consequently, for one column of synapse-device array, the V$_{output}$ can be simply expressed as below.

$$\begin{aligned} V_{output}= & {} (I_{array}-I_{shift}) \times R_{shift} = V_{array}-V_{shift}\\= & {} \sum \nolimits _{i=1}^{n}\{(V_{i}\times ({w_{ij}-w_{shift}}))\times R_{Load}\} \end{aligned}$$

Comparing to the typical expression of the negative weight (:pair-synapse method), we can minimize hardware resources by the weight shifter for realization of the negative weight, as shown in Fig. 2c, d^25,26^. Figure 2c shows a comparison of the number of additional devices, between the conventional pair-synapse method and the proposed weight shifter. When the synapse-device array size becomes larger, the number of additional devices increases exponentially for the pair-synapse method. In contrast, the weight shifter needs only a small number of additional devices because the size of weight shifter is only dependent on the number of rows in the synapse-device array (Fig. 2a). From this result, we can estimate the additional area for both methods, and shown in Fig. 2d. The ratio of area between both methods increased drastically, and it is obvious that a larger number of devices is required for the pair-synapse method.

$$\begin{aligned} Area\,ratio={Area\,_{pair\,synapse\,method} \over Area\,_{weight\,shifter}} \end{aligned}$$

**Figure 3:**
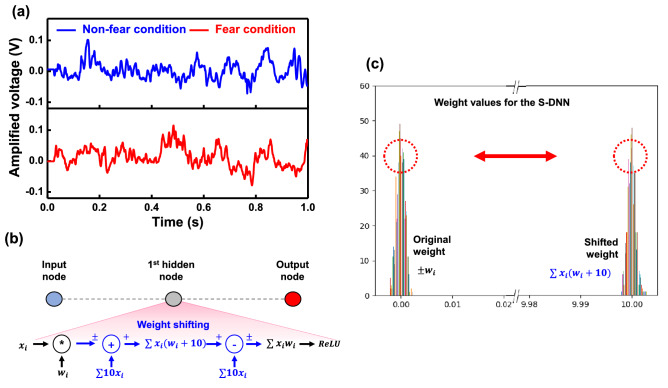
(**a**) Part of rat’s neural signals in non-fear and fear conditions. (**b**) Sequence of the weight shifter in S-DNN. Firstly, the weight values are positively shifted; then it returns back to origin before the ReLU function. (**c**) Weight values utilized in the S-DNN (original weights and shifted weights).

We constructed the S-DNN to demonstrate proposed weight shifter, as shown in supplementary Fig. S1. As an application, rat’s neural signals were recognized by the constructed S-DNN which has the weight shifter. Figure 3a exhibits parts of detected neural signals that were obtained in both fear and non-fear conditions. Detailed description of the neural signals is presented in the supplementary Figs. S2 and S3^27^, and the process of constructed S-DNN is simply described in Fig. 3b. From typical S-DNN, the weight shifting process was added; the VMM was conducted with a shifted median of weight. After the VMM, shifted values were returned back ($-\sum 10x_i$) when $x_i$ means input bias.

In other words, during both training and inference processes, the weight values remained positive because they were shifted as much as 10 towards the positive side. On completion of the VMM, the shifted values were returned back. All the original and shifted weight values utilized through the S-DNN are exhibited in Fig. 3c.

**Figure 4:**
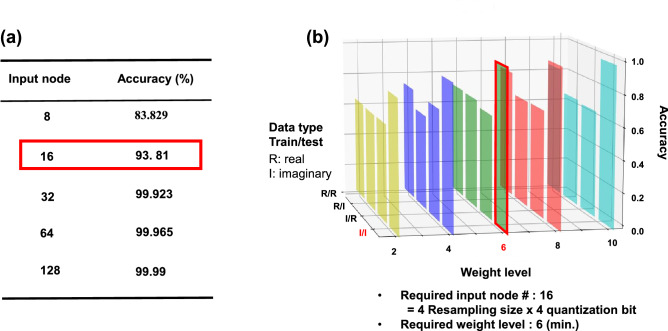
Optimized conditions of the S-DNN. For implementation of the H-DNN, the S-DNN was optimized to minimize hardware sources. (**a**) Input node dependence of recognition accuracy. For more than 90$\%$ of accuracy, at least 16 input nodes are required. (**b**) Optimization of various parameters such as data type, weight level, and input data preparation method (resampling size and quantization bit). More details about optimization of the S-DNN is described in the supplementary figures (Figs. S2–S4). For high recognition accuracy with minimized hardware source, 16 input nodes (4 resampling size and 4 quantization bit), imaginary input data, and 6 weight levels were utilized.

Moreover, the S-DNN was optimized to minimize the hardware resources for implementation of the weight shifter on a printed circuit board (PCB), as shown in Fig. 4. We compared various parameters such as the number of input nodes, number of weight levels, and types of data sets, besides the methods of input data preparation, viz., resampling size and quantization bit, details of which are explained in Figs. S3 and S4. The S-DNN was optimized for a high accuracy level (93.81%) with reduced hardware resources for the PCB level implementation, as shown in Fig. 4b (Figs. S2–S4). The higher number of input node showed higher recognition accuracy. However, for hardware implementation, minimum number of input node which can exhibit more than 90% accuracy was selected (Fig. 4a). There were also various dependence of input data type, resampling size, and quantization bit on the accuracy, as shown in Figs. S2–S4. To effectively minimize the hardware resources, we considered all above parameters (number of input node, input data type, resampling size, and quantization bit).

**Figure 5:**
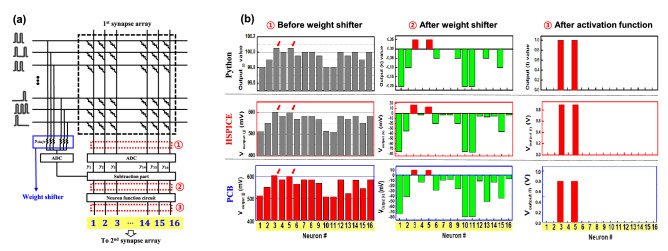
(**a**) Simplified schematic of realized H-DNN. To demonstrate the weight shifter, various output results (at 1 before weight shifter, 2 after weight shifter, and 3 after activation function) are compared by the S-DNN (Python), circuit simulator (HSPICE), and H-DNN (PCB). (**b**) The obtained output results were the same for all three cases: Python, HSPICE, and PCB.

Based on the optimized condition of Fig. 4, the weight shifter was evaluated and compared in three respects: S-DNN, circuit simulator, and PCB circuit (Figs. 5 and S5). The hardware implemented circuit having weight shifter is composed of input layer, 1st synapse arrays (16 $\times$ 16), weight shifter, neuron parts (analog to digital circuit (ADC), subtraction part, and activation function), 2nd synapse array (16 $\times$ 2), and output layer, as shown in Figs. 5a and S5.

As utilized bias, 16 input voltages which are prepared from rat’s neural signals are applied to the input layer (Fig. S2), and they are multiplied with conductance of the 1st synapse array. The results of multiplication: output currents (I$_{array}$) are converted to voltages (V$_{array}$), and the V$_{shift}$ is subtracted from the V$_{array}$ through the ADC and subtraction part. It implies the returning shifted weight back.

Consequently, the V$_{output}$ (output voltage of each column) is recognized at the activation function part which is constructed as a Rectified Linear Unit function (ReLU) circuit by comparator and integrator (Fig. S5c). When the V$_{output}$ exceeds a threshold voltage, the V$_{output}$ is applied to the next synapse array. In the other case, output voltage of the activation function part is suppressed to zero voltage. After these 1st synapse array and neuron parts, output voltages of the activation function part is applied to the 2nd synapse array (16 $\times$ 2).

In the same manner, applied voltages are multiplied with conductance of 2nd synapse array, then two output currents of the 2nd synapse array are converted to output voltages by the R$_{Load}$. These output voltages are compared each other at the end of neural network; one represents the fear condition and the other represents the non-fear condition.

Even though whole H-DNN was successfully implemented (Fig. S5d), we detected the output voltages at near the weight shifter to confirm whether the weight shifter can work well through the VMM. To evaluate the weight shifter, for all cases (S-DNN, HSPICE, and PCB), we compared the output values at three points: $\textcircled {\small 1}$ before weight shifter, $\textcircled {\small 2}$ after weight shifter, and $\textcircled {\small 3}$ after activation function. As described in Fig. S1, 16 $\times$ 16 synapse-device array was utilized to connect the input layer and hidden layer, which leads to 16 output values at the three points (highlighted 16 results of Fig. 5b).

Before the weight shifter, output values showed all positive values for all cases because the median of weight values was shifted to positive values. Then, through the weight shifter, the median of weight values was returned back to their original value, which led to the negative output values. Finally, after the activation function (designated as the ReLU), only the positive output values (number 3 and 5) remained. At the three points ($\textcircled {\small 1}$–$\textcircled {\small 3}$), all cases such as the S-DNN, HSPICE, and PCB exhibited the same results. This conclusively demonstrates effective transfer of the weight shifter function from software to hardware levels. Complete details about the hardware implementation are described in Fig. S5.

## Conclusion

Hardware implementation of the negative weight was realized in a simpler way by the proposed weight shifter. In comparison to the conventional pair-synapse method, the weight shifter minimized various hardware resources such as additional requirement of devices and power consumption, which resulted in less area-requirement and simplicity of circuits. To demonstrate the weight shifter, rat’s neural signals were recognized by the S-DNN, HSPICE, and PCB level H-DNN. During the recognition, employed weight values were positively shifted to be directly expressed by the conductance of the synapse device. For all cases (S-DNN, HSPICE, and PCB), the same output results were observed; this conclusively demonstrates that the proposed weight shifter can more effectively implement the negative weight with less hardware resources.

## Methods

To evaluate the proposed weight shifter, the S-DNN was constructed with 16 input nodes, one hidden layer, and 2–10 weight levels by using Python (Fig. S1). The employed weight values were positively shifted to confirm the weight shifter in the S-DNN. Through the constructed S-DNN, we recognized a rat’s neural signals in fear or non-fear conditions, as shown in Fig. S2. For the hardware implementation of the weight shifter, we optimized the S-DNN to reduce required hardware resources (Figs. S3, S4). Following this, the optimized S-DNN including weight shifter was realized as a circuit through a circuit simulator: HSPICE. For the weight shifter, 6.25 k$\Omega$ fixed resistors are employed as R1–R16. To express the conductance derived from the weight map of S-DNN (Fig. S5b), resistors (3.2–100 k$\Omega$) are utilized. In addition, op-amp (LM 338) was used to construct the ADC, activation function, and comparator. Finally, the developed circuit composed of the weight shifter, ADC, subtraction part, and transimpedance amplifier was implemented on the PCB (Fig. S5).

## Supplementary information

Supplementary Figures.
