# Supplementary material for: Alternative negative weight for simpler hardware implementation of synapse device based neuromorphic system
Source: Sci Rep. 2021 Dec 1;11:23198. doi: 10.1038/s41598-021-02176-4 (PMC8636521; doi:10.1038/s41598-021-02176-4)
Supplement: Supplementary file 1 — Supplementary Figures. [file 41598_2021_2176_MOESM1_ESM.pdf]

Alternative negative weight for simpler hardware implementation of synapse device based neuromorphic system

Geonhui Han[1], Chuljun Lee[1], Jae-Eun Lee[1], Jongseon Seo[1], Myungjun Kim[1], Yubin Song[1], Young-Ho Seo[1], and Daeseok Lee[1,*]

[1]Department of Electronic Materials Engineering, Kwangwoon University, Seoul 01897, Republic of Korea.
[*]leeds@kw.ac.kr

ABSTRACT

1 SUPPLEMENTARY MANUSCRIPT

1.1 Constructed S-DNN

In this research, S-DNN was constructed to evaluate the proposed weight shifter, as shown in Fig. S1. The S-DNN was consisted of three layers (input, hidden, and output layer) with 16 hidden nodes and 2 output nodes. For optimization of the S-DNN, various parameters, viz., number of input nodes, weight levels, types of data sets, and input data preparation methods were investigated. For the learning rule and activation function, back propagation learning rule and rectified linear unit (ReLU) function were utilized.

1.2 Input data preparation

In Fig. S2, the neural signals from fear and non-fear conditions were prepared as input data set. Fig. S2(a) shows raw data of the neural signals for fear and non-fear conditions[1]. Because the neural signals are noisy and complicated, it needs to be transformed as input data set (Fig. S2(b-c)). The fast Fourier transform (FFT) was utilized to transform the raw data from time domain to frequency domain. After the FFT, we defined resampling size and quantization bit to allocate data set. The resampling size and quantization are represented by the x-axis and y-axis, respectively, in the frequency domain result (Fig. S2(d)). From these parameters, the number of input nodes was derived. Note that the S-DNN was optimized to reduce hardware resources during the hardware implementation. Thus, minimum input nodes derived from resampling size and quantization bit were utilized with high recognition accuracy.

1.3 S-DNN optimization

Based on the constructed S-DNN, we conducted hardware implementation of the weight shifter, as shown in Fig. S3-5. During the hardware implementation, the S-DNN was optimized to reduce hardware resources because of the circuit complexity. Various parameters such as number of input nodes, weight levels, type of data sets, resampling size, and quantization bit were evaluated (Fig. S3 and S4). Considering the number of input nodes (both resampling size and quantization bit), higher number of input nodes indicated higher recognition accuracy: higher weight level also leads to higher recognition accuracy. There was also dependence on the type of data set, and all of these indicate that a large number of input nodes and high weight levels could lead to the best accuracy but also results in circuit complexity. Thus, we found that the conditions that could minimize the hardware resources and circuit complexity as in the following list: 16 input nodes including 4 resampling size and 4 quantization bits, 6 weight levels, imaginary data sets for both train and test.

1.4 Hardware implementation

For the hardware implementation at the PCB level, the results of optimized S-DNN were utilized, as shown in Fig. S5. Simplified circuit of the H-DNN is illustrated in Fig. S5(a); the input layer and hidden layer were connected to each other through the first synapse device-array (16×16). The hidden layer and output layer were connected through the second synapse-device array (16×2). The prepared input data set (from the neural signals) was expressed as input pulses and the weight values of synapse device array were realized as equivalent circuit based on the weight map extracted from the trained S-DNN (Fig. S5(b)).

Based on the input pulses which represent neural signals, the synapse array could lead to VMM and result in summed current for each column. This current is converted to a voltage by a transimpedance amplifier and digitized through an analog to digital converter (ADC)[2]. After that, the subtraction between results of the ADC and weight shifter is conducted at a subtraction part. The results are then applied to the second synapse-device array through the activation function part, which is designated as the ReLU function circuit. Before implementation at the PCB level, the entire H-DNN circuit developed by us was confirmed by HSPICE, as shown in Fig. S5(c). Finally, the H-DNN with the weight shifter was implemented on the PCB, as shown in Fig. S5(d).

References

1. Lee, J. H., Lee, S. & Kim, J.-H. Amygdala circuits for fear memory: a key role for dopamine regulation. *The Neurosci.* **23**, 542–553 (2017).

2. Yeo, I., Chu, M., Gi, S.-G., Hwang, H. & Lee, B.-G. Stuck-at-fault tolerant schemes for memristor crossbar array-based neural networks. *IEEE Transactions on Electron Devices* **66**, 2937–2945 (2019).

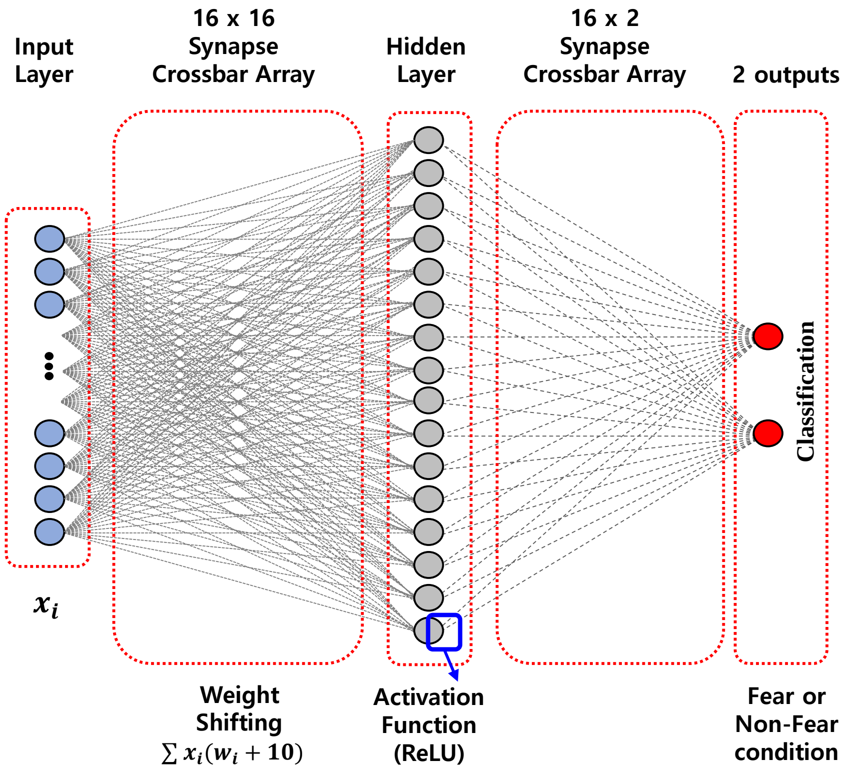

Figure 1. (a) Schematic of constructed S-DNN.

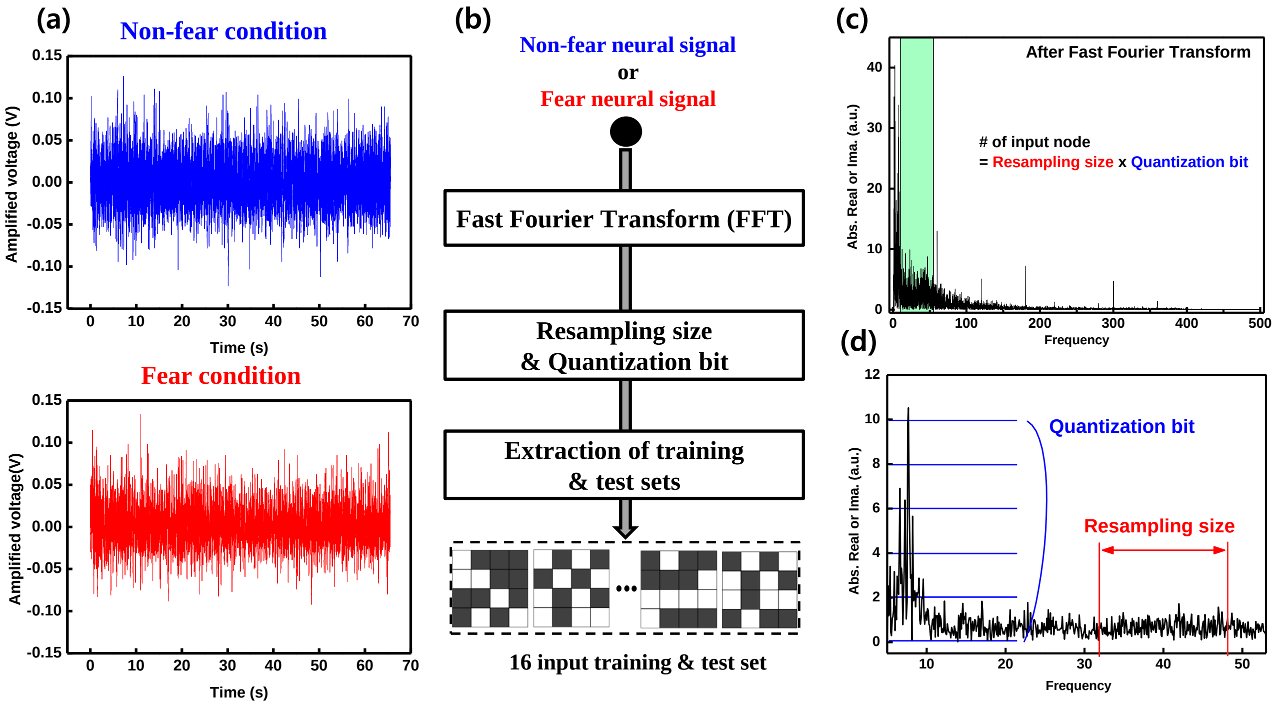

Figure 2. (a) Rat's neural signals in non-fear and fear conditions for whole measured time. (b) Sequence of input data preparation. (c) After the FFT, resampling size and quantization bit were allocated. (d) Enlarged plot of green region.

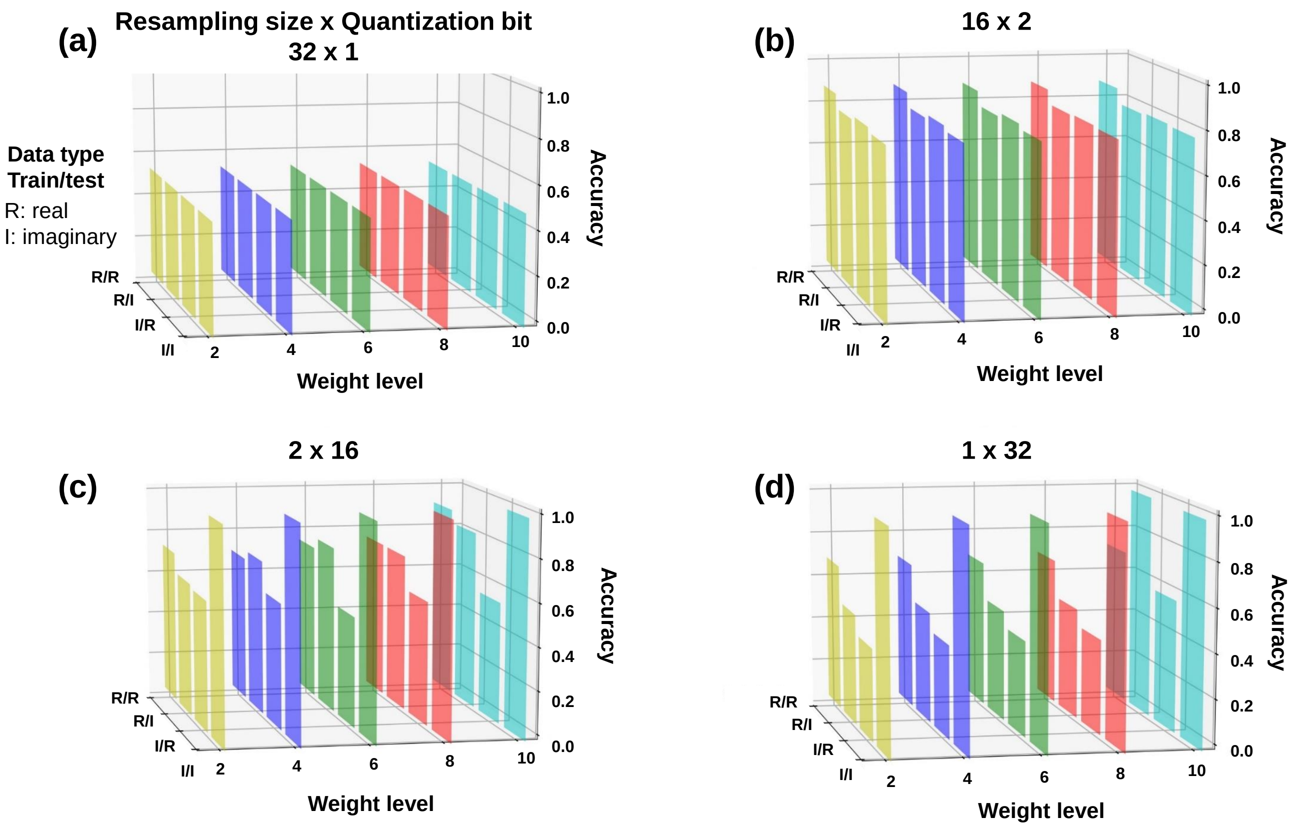

Figure 3. Recognition accuracy results for 32 input nodes case. The resampling size and quantization bit were controlled from one to 32. (a) 32 resampling size × 1 quantization bit (b) 16 resampling size × 2 quantization bits, (c) 2 resampling size × 16 quantization bits and (d) 1 resampling size × 32 quantization bits.

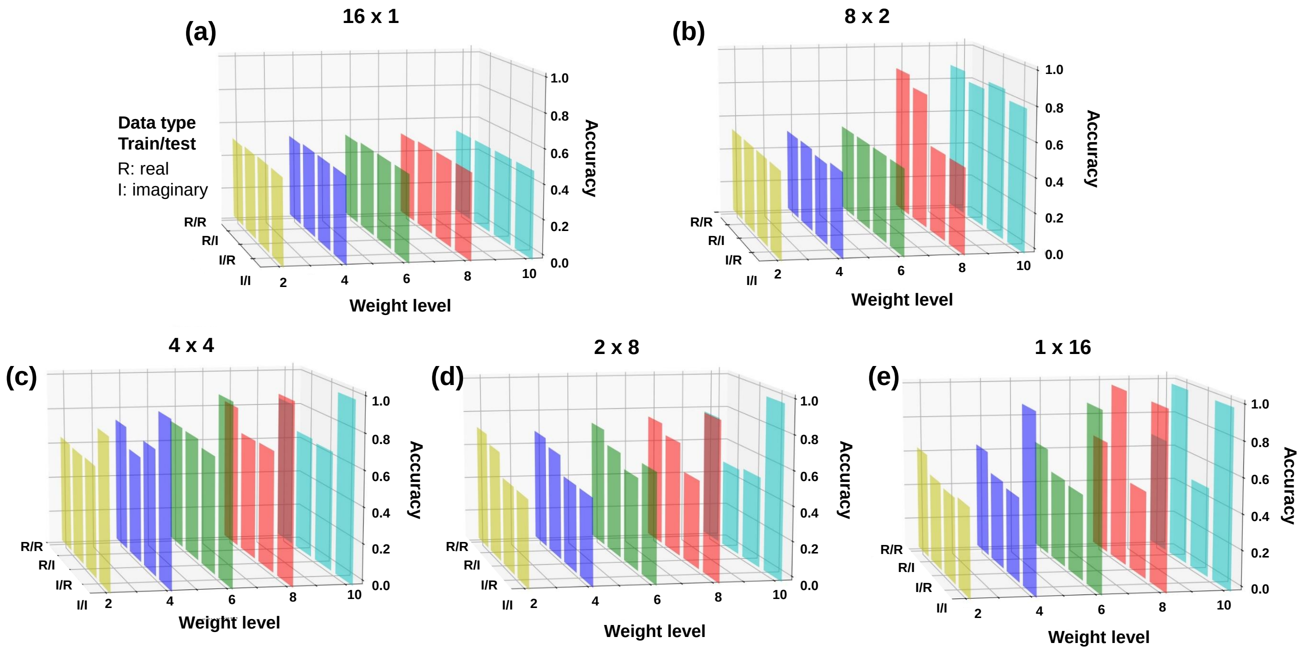

Figure 4. To minimize the hardware resources, less input node case was also evaluated. For 16 input nodes, the resampling size × quantization bit was respectively (a) 16 × 1, (b) 8 × 2, (c) 4 × 4, (d) 2 × 8, and (e) 1 × 16. Considering the high recognition accuracy and number of hardware resource, 4 × 4 case was the optimized condition.

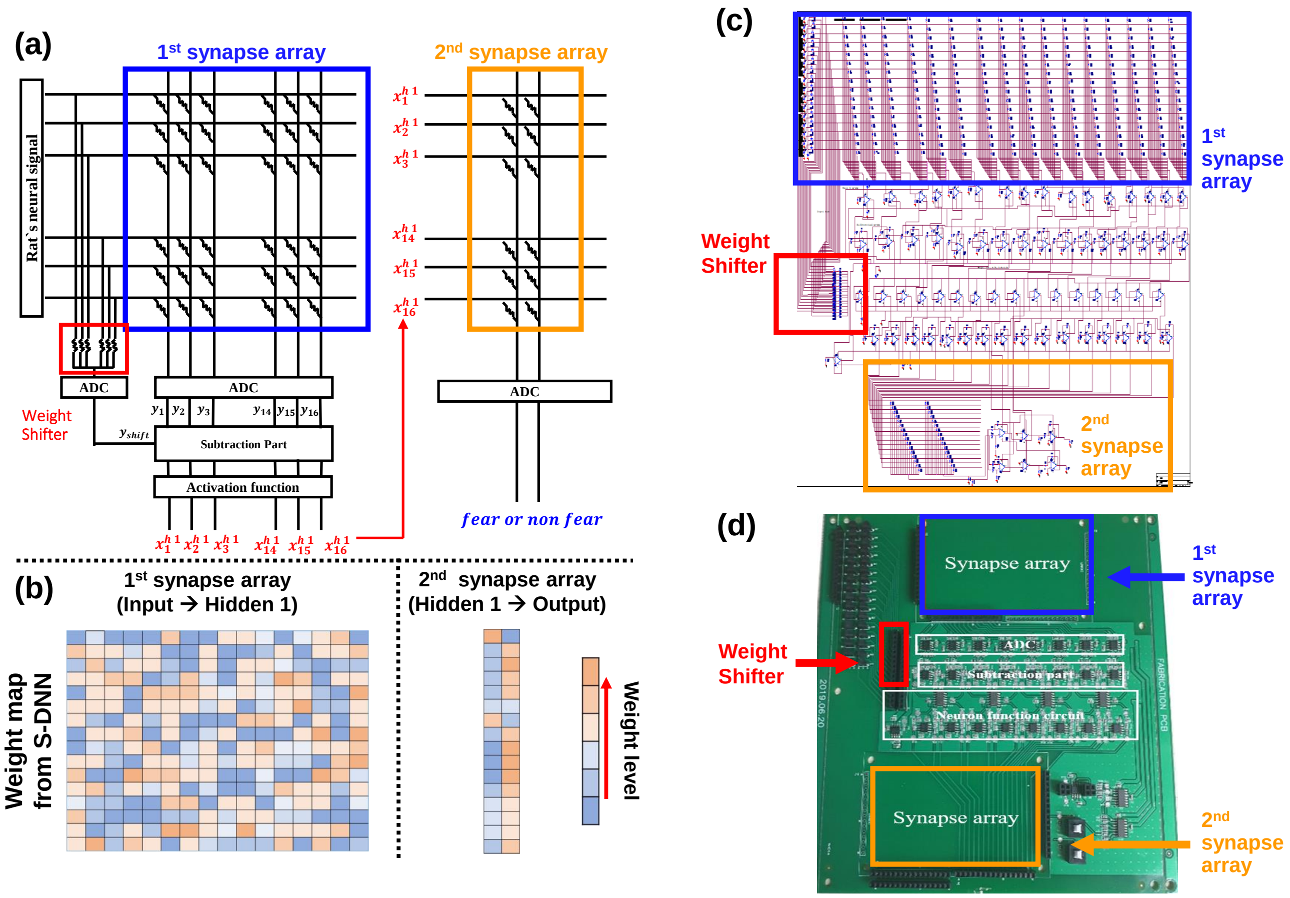

Figure 5. (a) Simplified schematic of the H-DNN with the weight shifter. (b) Extracted weight map used in the S-DNN. Each level of weight map can be expressed as conductance level of synapse device. (c) The developed H-DNN circuit confirmed by HSPICE. (d) A photograph of the H-DNN including with the weight shifter.
